# Indents

**Published:** 1909-09-15

**Authors:** 


### INDENTS.

**☞Brief Articles of Technical or Scientific Value will receive notice and comment. Contributions invited.**

*Hygienic Education* Why speak of boiling each glass of water to destroy a few harmful bacteria, while a neglected and decayed tooth contains thousands? Teach our women that their mouths should receive quite as much attention as their finger-nails, with a far more salutary effect. Our greatest duty in this line of work is to enlighten the physician, to teach him that many of the stomach and intestinal troubles would have been prevented if there had been perfect mastication and cleanliness of the mouth, and explain to him that many harmful bacteria enter the system through the tonsils and fauces.

We must impress on the public that the mouth is the greatest germ-breeding pen of the human body, and that many of the contagious, infections, or germ producing diseases are established through germs produced and multiplied in the mouth. The medical mind and the public have never been so receptive to this teaching as they are now. Let us get busy. Dental associations might approve articles on these subjects and encourage the profession to give them to our patients. Encourage the reading of such articles in schools, churches and societies where they will be well received.—C. C. Harris in *Cosmos*.

*Thermal and Electric Conductivity of the Principal Filling* The physical and chemical properties of the various filling material have been subjected to scrutinizing tests by various authors. The present investigation recommends itself by the thoroughness of the thermal tests and the extensive statistics procured. The thermal conductivity of the filling materials was tested first by itself and then under conditions in filling approximating the normal. The first series of tests consisted in exposing small cylinders of 8 mm. diameter and 10 mm. height to a constant heat of 100 degrees from a specially designed boiler, the upper surface of these cylinders having been covered with wax, and the time necessary for the melting of this wax being registered by a chronograph. These tests were repeated ten times with each material, the average time necessary for the melting of the wax being as follows: Silver amalgam 9.24 seconds, copper amalgam 9.17 seconds, silicin cement 98 seconds, Ascher cement 102 seconds, Harvard cement 108 seconds; the gutta-percha cylinders became soft to half of their height in about two hours, the wax on the surface did not melt. In testing the thermal conductivity of filling materials under conditions in the tooth itself approximating the normal, a molar with an occlusal cavity was embedded in an iron cup filled with mercury up to the level of the filling, and exposed to the same heat as the specimens in the first series. In the fillings an iron pin was inserted in order to conduct the heat, and the pin mounted with a pellet of wax. For the first series of tests, which were repeated five times with each material, the pin extended 1 cm. above the surface of the filling, the average time required for the melting of the wax being for cohesive gold 13.5 seconds, soft gold 16.7 seconds, gold inlays 16.4 seconds, silver amalgam 19.6 seconds, copper amalgam 17.6 seconds, silicin cement 31.6 seconds, Harvard cement 30.4 seconds, Ascher cement 36 seconds. In a second series, in which the pin was extending only 2 mm. above the surface of the filling, the results were as follows: Cohesive gold 4.25 seconds, soft gold 5 seconds, gold inlay 7.8 seconds, silver amalgam 6 seconds, copper amalgam 9.25 seconds, silicin cement 15.5 seconds, Harvard cement 9.5 seconds, Ascher cement 17.5 seconds. Another series of experiments was carried out without a pin, the wax being laid on the surface of the filling itself. The results were as follows: Cohesive gold 15.4

seconds, soft gold 13.4 seconds, gold inlay 25.4 seconds, Harvard cement 28.4 seconds, Ascher cement 37.7 seconds, silicin cement 29.6 seconds. Another series of 8 experiments yielded for gold inlays without cement an average of 21.16 seconds, for inlays with cement 24.62 seconds.

The test as to electric conductivity were carried out with a galvanometer of the Deprez d'Arsonval system, and showed that cements in a dry state have an insulating action, which is slightly impaired if the cements are wet; gutta-percha is also an insulator. In order to test the metallic materials the amalgams were packed in a glass tube of 4 cm. length and of 2½ cm. in its lumen; contact was secured by two copper points, and a Thomson double bridge for measuring very feeble resistances was employed. The results were the following: Silver amalgam 0.00282 ohm, copper amalgam 0.00123 ohm. The conductivity of the amalgams approaches that of the pure metals, lying between that of copper and mercury. The copper amalgam tested was found to be approximately twice as good an electric conductor as the silver amalgam.—By Christo Kousleff in June *Cosmos*.

*Canker Sores.* The ulcers generally known as canker sores are among the most common pathological conditions of the mouth, if we except the diseases dependent upon the teeth. These ulcers are generally single, though occasionally there may be two or more at the same time. They seem to appear suddenly and are quite persistent unless given suitable treatment. Their site is most commonly at the duplicature of the mucosa of the cheek and the gums, though they are occasionally seen on the floor of the mouth and on the edges and under surface of the tongue. They vary in size from that of a grain of wheat to that of a small sized bean. In shape somewhat lenticular or oval. Their depth varies, but can never be considered superficial. Their margins are rather well defined, but not so markedly as are chancrous ulcers of similar tissues, neither are they so irregular as are lupus ulcers of the mouth. The mucosa for a quarter to a half inch from the ulcer is of a deep red colcr. The base of the ulcer is overlaid with a grayish white necrotic covering, not unlike that found in syphilitic ulcers in the mouth. When this coating is removed, a granulating surface is exposed, which is extremely sensitive to touch, but bleeds but slightly, if at all. The lympathtics doing police duty over the area involved do not, so far as my observation goes, become involved. These ulcers are so distinctive in their appearance that they can hardly be mistaken for any other lesion.

As to the nature and etiology of canker sores, there has been, and still is, some difference of opinion. Pusey and other prominent dermatologists believe them to arise from trophic disturbances and class them under the head of herpes simplex. That herpatic ulcers of the mouth are in some way associated with gastro-intestinal disturbances there seems little doubt, since they seem to accompany or follow gastric attacks in persons predisposed to herpes. Slight trauma may be the exciting cause.

Local treatment of the herpetic ulcers of the mouth is generally curative. If they are touched with silver nitrate, they respond quickly. If the ulcer is cleansed with peroxide of hydrogen, then dried, and a 20 per cent. solution of zinc chloride applied, a cure usually follows one application.

Herpes labialis or cold or fever sores, as they are known by the laity, have a similar origin. They differ in appearance principally on account of location and tissue involved. Herpes of the lips and occasionally of the gums, sometimes follow dental operations and cause uneasiness on the part of the patient, he attributing this condition to infection from unclean armamentarium used by the dentist, when, in truth, the patient is of a herpetic diathesis, and under such circumstances a slight irritation only, in such locations. being sufficient to excite the condition. Herpes labialis and herpes gingivalis in character are much more alike than are herpes labialis and canker sores. Those of us who have practiced in malarial districts know that patients suffering from what is known as chills and fevers are very subject to herpes labialis; the toxic elements accompanying, seemingly, are prone to cause herpes at the muco-cutaneous junctures about the mouth.

Herpes gingivalis is best treated by thorough cleansing with peroxide of hydrogen and painting with compound tincture of benzoin. A mouth wash, composed of a saturated solution of boracic acid to which one drop of the oil of cassia to the ounce of the solution is added, may be used to supplement the peroxide and benzoin. Herpes labialis is benefitted by the application of tincture of camphor or alcohol and while yet moist from the application, a coating of boracic acid powder may be dusted on, which acts as a dryer and protector. In case of cracks in the lips, collodion serves to immobolize.—Dr. T. L. Gilmer in *Review*.

*The Boston Conference.* In his benediction to the Boston Conference on Oral and Dental Hygiene, which ended on the twenty-third day of January at Huntington Hall, President Elliott said, "There is coming a great change in the practice of medicine and surgery and dentistry, and a much greater proportion of the attention of these professions is hereafter to be devoted to prevention rather than cure."

Dental Hygiene is an important part of a broad, and universal movement for better care of the public health through a more complete knowledge of the conditions which promote health. The dentist must now take his active part and responsibility in the great progress for preventive medicine which is everywhere working. He must give to it his skill, his time, his money.

One of the best and surest marks of civilization is found in the attitude of its medical men—and the dentist belongs to the medical profession; and those medical men who consider themselves simply a kind of glorified mechanic remain in a purely shop point of view.

To no group of men and women is given a larger opportunity to render real human service than falls to the dentist and the dental nurse. Through the practice of their knowledge and service life can be made more effective, more useful and fuller. It can be lengthened in years; it can be given more enjoyment and pleasures; and many of the obstacles which prevent clean living can be levelled.

Even the meagre dental inspection which has been made has been most significant. It has thrown a flood of light and a little deeper into the problems of home and school hygiene. Facts are being discovered—great truths the momentous bearing of which upon life and health are new to all thoughtful people. One more layer of symptom is bored through to the foundation of cause where tinkering and cure must give way to prevention.

The parent, the teacher, the social worker, the nurse, the physician—the public are looking to the dentist to meet and do his clear duty. Every dentist who has the true professional spirit will measure up to his duty; and being a man, he will give substantial aid willingly and generously, according as it is his privilege, in helping his fellow men profit by his knowledge and skill.

No form of practical philanthrophy ever had so fair and favorable a beginning. Dental clinics can be established on a proper foundation, the patient paying a normal charge for service received. Hospital boards and social workers are ready to co-operate with dentists in establishing and maintaining these clinics. Medical inspection of school children has been organized and put into practice; and school authorities are ready to include the examination of the teeth in school inspection, and provide instruction upon the care of the mouth and teeth. Dental and medical schools are beginning to instruct their students in oral and dental hygiene, and the important part it has in helping to increase and maintain public health. The Dental School of Harvard University has been given one thousand dollars, and a promise of more, to carry on research work in preventive dentistry; and a working fellowship has been created. In the free popular lectures on medical subjects offered to the public by medical schools are lectures on dental hygiene. In the crusade against tuberculosis oral and dental conditions play an important part. The spread of infectious diseases through the neglect of the mouth and teeth is a subject of growing importance.

All these broad and human activities are distinctly within the dentist's sphere; and his responsibility and the part he is to play in the prevention of disease and the prolongation of life stand in his path of public duty. One result of the Boston Convention is that organized bodies of workers, social, charitable, educational and professional stand waiting for him to answer the "*Call to Public health*"—a call which clearly summons the dentist.—Wm. R. Woodbury, in *The Journal.*

*Value of Association.* There is or used to be, an opinion among some of our religious people that to find one's self and get near to the Creator one had to go into solitude in order to study and examine one's self carefully and closely, but it is this idea that we should find ourselves in society work, that struck me as being one of the most vital incidents that we could take away from this meeting. We all know, especially those of us who are teachers in colleges, that most students have a very meager conception of what it means to be a professional man. It is the constant effort and endeavor of every conscientious teacher in a school to try to develop the professional man in the student before him. One of our ablest and best known dental editors when I asked him recently what it was that kept him at his work of strenuously providing copy for his journal, month after month, year after year, said "it certainly was not the salary, but it was the thought that he was doing something to instill into the mind of the professional in general professional ideas." It is the thought of the ideal which the teacher, the preacher, the editor, the essayist, and the speaker who has preceded me, have had in mind; they want their ideals developed and crystalized, so to speak, into professionalism. What is it to be a professional man? Our students come to us with no true idea. I generally ask every student when he first presents himself why he studies dentistry; the answer in a large majority of cases is that it is a respectable profession, and it looks to him as if in it there was a good opportunity to make a living. That is all very well, and we do not want to discredit a man for entertaining any such ideas, but that is not the noblest thought a man could have; it is not the real goal, and it is not the professional one. The ideal that we ought to have is that which will give to us a true spirit of professionalism, and as I have thought on this subject I have wondered how long it would take a man who sits down in his own office and makes no effort to develop a professional spirit and have professional ideas to develop them if he never comes in contact with other professional men. How long would it take him to develop a high ideal in this way? In other words, how long would it take a man to find himself? It is true that some men will develop much more rapidly than others, but I believe that it is in this associated effort that most of us very much sooner come to find ourselves than in any other way. I believe that this the true feeling and experience which comes from the associating together of dentists. My experience has been that there are a few men in the profession who are expected to take up most of the time discussing the society papers so that there seems to be little opportunity for discussion by diffident and younger men, and opportunity for them to find themselves by participating in the discussions is lost. As Dr. Callahan has said, this is evidence in reading the proceedings of any of our dental societies. Most men hesitate, not because they do not know, or have good thoughts, but because they are modest—may I say that they are overly modest, or feel that they cannot instruct any one—so they do not say anything. It is not always, it seems to me, that the oldest or most experienced man, gives the best instructions. Some things that we say, and we see them when put in print, do not seem to contain great wisdom, and they may even seem absurd and foolish, but the earnestness and the manner of the speaker has a great influence that is incalculable in results because it may set some one to thinking. As I view my own professional life, looking back over its history, I find what it was that has inspired and stimulated me. It has unquestionably been the men with whom I have been associated. They gave me all the professional insight 'that I have ever obtained. It has not come from the things they said to me, but from the manner in which they said these things, and the earnestness with which they brought these matters to my notice. The numerous men that I have known in my professional career have had a wonderful influence upon my life, and it seems to me that I owe so much to these men that I can never repay it except by doing what I can to pass it on and make others happy. When I read the thoughts of other men, I read a great deal written by men that I have never seen, but their influence has been nothing to compare with that of the men I have known in person, and with whom I have discussed the problems of professional life, or that I have seen face to face and that I have been privileged to take by the hand and have a cordial and friendly greeting, while they have given me helpful words of encouragement. It seems to me that this is one of the greatest influences of association work and that it does a great deal towards developing our professional careers and instincts. I believe, truly, that nothing can take the place of the Dental Association or convention. Dental conventions are numerous now-a-days, and it sometimes seems as though we were overdoing the convention idea. There is to-day, in session in the city of Chicago, The American medical Association with an attendance of fifteen thousand physicians and dentists from all parts of our country. There are conventions of various kinds all over this country at this time of the year, and the amount of money and time that is spent in attending them would print a lot of books which could be distributed to every man who is interested in dentistry in this country, and might bring all known knowledge to him, and he might learn the things he ought to know. But I doubt whether all the money spent in this way could be spent to as good advantage as the money we shall spend in attendance upon this convention. This is a grand opportunity to look into each others' faces and to find out what each of us is doing, to get the word of cheer, help and comfort that we can get in no other way—not even from books or professional periodicals. I feel as though the one thought I want to emphasize in this discussion is that the opportunity which the dental convention affords to the young man to find himself, or the old man either, can nowhere else be realized so completely as in associated efforts of earnest and faithful professional men.

As a means of inspiration it is invaluable in many ways. When you attend dental associations your patients, your friends and acquaintances all know it and they will soon notice that you have been somewhere. You have lighted a new torch; you will do your work better and with more enthusiasm if you attend the meetings.

I want, also, in this connection, to say something about the small society or association. I know of several small cities where organizations have been formed recently, and the whole community, so far as professional interests are concerned, has been revolutionized; where before the dentists were merely speaking to each other on the street, they are now good fellows, they meet each other socially, they meet in society work more often. They stop and converse with each other on the street They have stopped saying mean and contemptible things about each other and have created a purer atmosphere and are happier, as well as more prosperous, and their people are better served.

I know one community that is now completely disorganized professionally because the dentists in that city refuse to have anything to do with each other. Those men are no longer professional; they have lost their way; they are groping in the dark, and it is a pity that they have gotten into such a condition as that, it is bad for the community.

Let us encourage this association idea and get into the habit of attending conventions, such as we are having here to-day and such as are being held in Chicago and many other smaller places. If there are no more than three or four men in a village they should get together and organize an association; dine and visit, do anything in a social way that will bring you nearer together, talk professional affairs and find out what each one is aiming at, and if some are aimless get them interested in something of a professional character, and I am sure in this way we can help our neighbor to hold his place, and we shall all get a greater conception of duty.—N. S. Hoff in *Dental Summary.*

